# When a Contaminant Is Not a Contaminant: A Case Report of Micrococcus luteus Causing Septic Shock in the Absence of Traditional Risk Factors

**DOI:** 10.7759/cureus.118562

**Published:** 2026-10-25

**Authors:** Jewairia Alajab, Mia K Sooch, Mohammed Yeddi, Omer Yeddi, Moayyed Hussein, Ahmed Yeddi, Benjamin Frimodig

**Affiliations:** 1 Department of Internal Medicine, Wayne State University School of Medicine, Detroit, USA; 2 Department of Internal Medicine, Sheikh Sultan Bin Zayed Hospital, Sharjah, ARE; 3 Faculty of Pharmacy, Cyprus International University, Nicosia, CYP; 4 Department of Internal Medicine, Detroit Medical Center, Wayne State University School of Medicine, Detroit, USA

**Keywords:** bacteremia, blood culture contaminant, micrococcus luteus, opportunistic infection, septic shock

## Abstract

*Micrococcus luteus* is a gram-positive, coagulase-negative coccus commonly regarded as a non-pathogenic commensal organism of human skin and is frequently considered a contaminant when isolated in blood cultures. However, emerging evidence suggests that it may act as an opportunistic pathogen in certain clinical settings. We report a case of septic shock caused by *M. luteus* in a 47-year-old woman with a history of hypertension and type 2 diabetes mellitus who presented with altered mental status and hemodynamic instability. Despite the absence of indwelling devices, prosthetic material, or structural heart disease, multiple blood cultures were positive for *M. luteus*, confirming true bacteremia. The patient required aggressive fluid resuscitation, vasopressor support, and intravenous antibiotic therapy, with subsequent clinical improvement and clearance of bacteremia. This case highlights the importance of correlating microbiological findings with clinical presentation and underscores the potential pathogenicity of organisms traditionally considered contaminants.

## Introduction

*Micrococcus luteus* is an aerobic, gram-positive, coagulase-negative coccus that colonizes human skin and mucosal surfaces and is generally considered a nonpathogenic organism. In clinical practice, it is most often encountered as a contaminant when isolated from blood cultures [1].

Despite this, *M. luteus* has been reported to cause invasive infections, including bacteremia and endocarditis, most commonly in immunocompromised patients or in association with indwelling devices or prosthetic material [2]. Clinically significant infection in immunocompetent individuals without these risk factors is uncommon, and progression to septic shock is rarely described [3]. This can complicate interpretation of positive cultures and delay recognition of true infection.

We present a case of septic shock attributed to *M. luteus* in a patient without indwelling devices or structural heart disease, highlighting its potential pathogenic role and the importance of clinical correlation.

## Case presentation

A 47-year-old woman with a history of hypertension and type 2 diabetes mellitus presented to the emergency department with a two-day history of altered mental status. According to family members, she exhibited confusion and was unable to recognize familiar individuals. She denied fever, chills, nausea, vomiting, cough, shortness of breath, abdominal pain, dysuria, or recent medication changes. 

Medical history was significant for hypertension and type 2 diabetes well managed with amlodipine and metformin, respectively. Hemoglobin A1C of 6.8% was noted three weeks prior to the present illness.

On presentation, her temperature was 38.1°C, heart rate 121 beats per minute, and blood pressure 80/45 mmHg. Physical examination revealed a confused but arousable patient without focal neurological deficits. There were no signs of skin breakdown, indwelling catheters, or sources of infection on examination.

Initial laboratory studies demonstrated leukocytosis with a white blood cell count of 22,000/µL, including 90% neutrophils. Urinalysis was unremarkable and random blood glucose was 102 mg/dL (Table 1). Chest radiography was unremarkable (Figure 1). Given the presence of hypotension, fever, and altered mental status, septic shock was suspected.

**Table 1:** Summary of laboratory values on admission

Parameters	Patient Value	Reference Range
WBC count	22 × 10^9^/L	4.5-11 × 10^9^/L
Neutrophils	90%	40-70%
Platelet count	210 × 10^9^/L	150-400 × 10^9^/L
Hemoglobin	13 g/dL	12.0-17.5 g/dL
Hematocrit	40%	36-52%
Blood glucose (random)	102 mg/dL	70-140 mg/dL
Serum lactate	2.9 mmol/L	0.5-2.2 mmol/L

**Figure 1:**
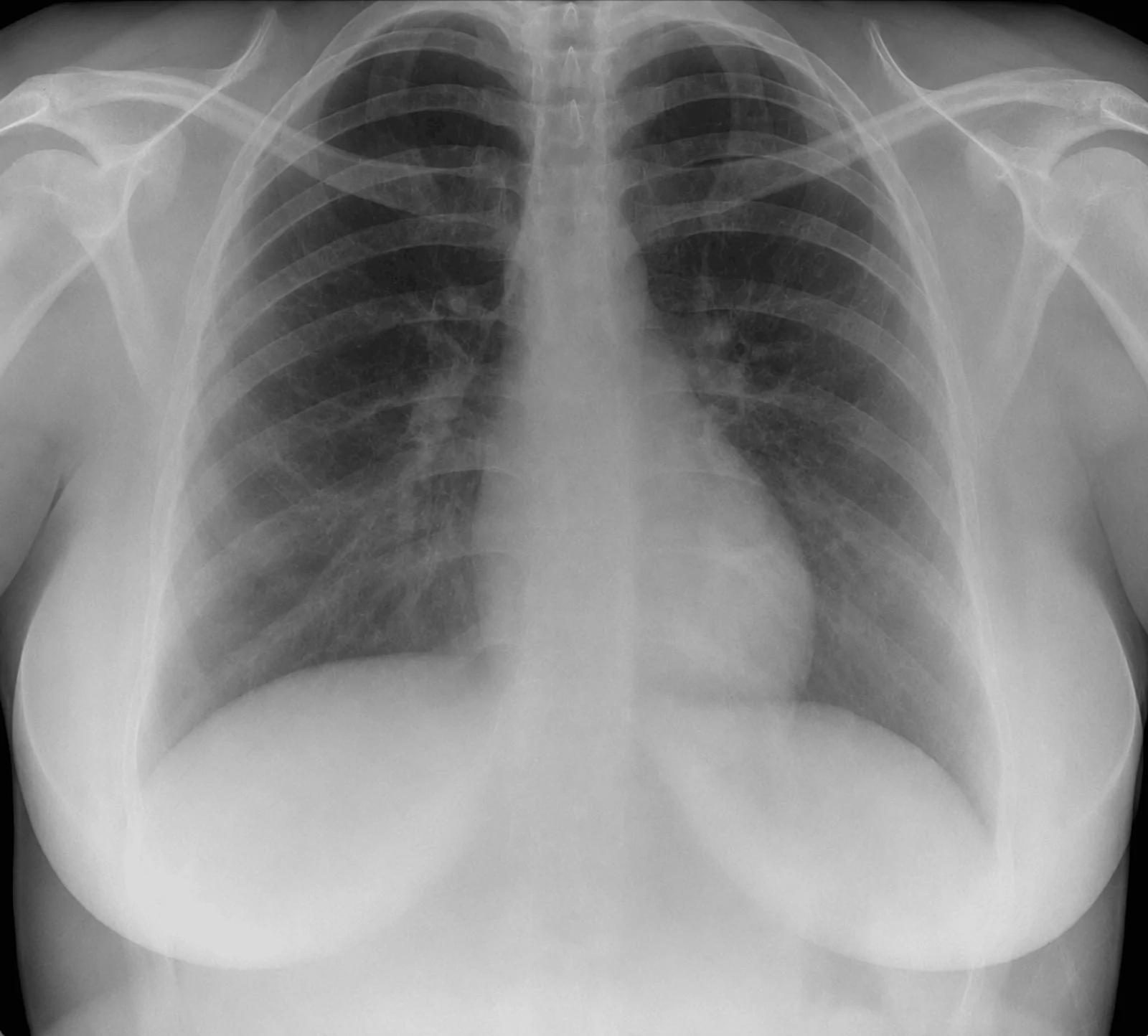
Normal chest radiograph findings on presentation

The patient received aggressive intravenous fluid resuscitation; however, she remained hypotensive after 4 L of crystalloid administration and required transfer to the intensive care unit. A norepinephrine infusion was initiated, resulting in improvement in mean arterial pressure. Empiric broad-spectrum antibiotic therapy with intravenous vancomycin and cefepime was started after obtaining blood cultures.

Blood cultures obtained from two sites subsequently grew gram-positive cocci in clusters. Antibiotic therapy was narrowed to vancomycin (Table 2).

**Table 2:** Summary of antibiotic susceptibility results

Antibiotic	Zone Diameter (mm)	Interpretation
Penicillin (P)	<15 mm	Resistant
Vancomycin (VA)	≥17 mm	Sensitive
Tetracycline (TE)	≥19 mm	Sensitive
Ciprofloxacin (CIP)	≥21 mm	Sensitive

All four blood culture bottles ultimately grew *M. luteus*, confirming true bacteremia rather than contamination. The patient had no history of valvular heart disease, prosthetic devices, recent invasive procedures, or indwelling catheters. Transthoracic echocardiography showed no evidence of valvular vegetations, and HIV testing was negative. 

The patient demonstrated clinical improvement with antibiotic therapy and vasopressor support, allowing discontinuation of vasopressors. She completed a 10-day course of intravenous vancomycin. Repeat blood cultures drawn on day 7 of hospitalization were negative, and her symptoms resolved prior to discharge.

## Discussion

Most reported cases of *M. luteus* bacteremia occur in immunocompromised patients or in association with indwelling medical devices [4]. In contrast, our patient had no prosthetic material, indwelling catheters, or structural cardiac abnormalities. The present case is noteworthy for several reasons. First, the patient was immunocompetent; however, type 2 diabetes mellitus may have contributed to susceptibility through impaired phagocytic function and hyperglycemia-mediated immune dysfunction [5]. 

Second, the medical history did not reveal classical risk factors, such as indwelling catheters, prosthetic devices, or valvular heart disease [6]. Third, the patient developed septic shock, with a qSOFA score of 3, a manifestation rarely associated with *M. luteus* [7]. 

A key challenge in this case was distinguishing true bacteremia from contamination. The presence of *M. luteus* in all four blood culture bottles, in conjunction with the patient’s clinical presentation of septic shock and absence of an alternative infectious source, strongly supported a diagnosis of true infection. Additionally, the patient’s clinical improvement following targeted antimicrobial therapy further reinforces the pathogenic role of the organism. 

Management of *M. luteus* bacteremia is not well standardized due to its rarity. The organism is generally susceptible to a range of antibiotics, including vancomycin and beta-lactams [8]. In this case, the patient responded well to vancomycin therapy, with resolution of bacteremia and clinical recovery. 

*M. luteus* is traditionally considered a non-pathogenic commensal organism and is commonly regarded as a contaminant when isolated in blood cultures. However, increasing evidence suggests that it may act as an opportunistic pathogen capable of causing clinically significant infections [3]. 

This case emphasizes the importance of correlating microbiological findings with the clinical picture and highlights that organisms typically dismissed as contaminants may, in certain contexts, represent true pathogens capable of causing severe disease. 

## Conclusions

Although *M. luteus* is commonly considered a contaminant, it can cause severe infections, including septic shock, even in patients without traditional risk factors such as indwelling devices or prosthetic material. Multiple positive blood cultures and concordance with the clinical presentation should prompt consideration of true bacteremia. Early recognition and appropriate antimicrobial therapy are essential to ensure favorable outcomes.
